# Invasive Cutaneous Melanoma: Evaluating the Prognostic Significance of Some Parameters Associated with Lymph Node Metastases

**DOI:** 10.3390/medicina59071241

**Published:** 2023-07-03

**Authors:** Octavia Vița, Aura Jurescu, Adrian Văduva, Remus Cornea, Marioara Cornianu, Sorina Tăban, Diana Szilagyi, Cristian Micșescu, Bianca Natarâș, Alis Dema

**Affiliations:** 1 Department of Microscopic Morphology-Morphopatology, ANAPATMOL Research Center, “Victor Babeș” University of Medicine and Pharmacy, 300041 Timișoara, Romania; 2 Department of Pathology, “Pius Brînzeu” County Clinical Emergency Hospital, 300723 Timișoara, Romania

**Keywords:** melanoma, Breslow thickness, mitotic rate, ulceration, Clark level, lymph node metastases

## Abstract

*Background and Objectives*: This study aimed to assess the clinical-pathological profile of patients with invasive cutaneous melanomas and to identify the parameters with a prognostic role in the lymph nodal spread of this malignant tumor. *Materials and Methods*: We performed a retrospective study on patients with invasive cutaneous melanomas who underwent surgery in the “Pius Brînzeu” County Clinical Emergency Hospital from Timișoara, Romania, and were evaluated for the status of loco-regional lymph nodes. We selected and analyzed some parameters searching for their relationship with lymph node metastases. *Results*: We identified 79 patients with invasive cutaneous melanomas (29 men and 50 women, mean age 59.36 years). A percentage of 58.3% of melanomas had Breslow tumor thickness >2 mm; 69.6% of melanomas showed a Clark level IV–V. Tumor ulceration was present in 59.5% of melanomas. A mitotic rate of ≥5 mitoses/mm^2^ was observed in 48.1% of melanomas. Tumor-infiltrating lymphocytes (TILs), non-brisk, were present in 59.5% of cases and 22.8% of patients had satellite/in-transit metastasis (SINTM). Tumor regression was identified in 44.3% of cases. Lymph nodes metastases were found in 43.1% of patients. Statistical analysis showed that lymph node metastases were more frequent in melanomas with Breslow thickness >2 mm (*p* = 0.0002), high Clark level (*p* = 0.0026), mitotic rate >5 mitoses/mm^2^ (*p* = 0.0044), ulceration (*p* = 0.0107), lymphovascular invasion (*p* = 0.0182), SINTM (*p* = 0.0302), and non-brisk TILs (*p* = 0.0302). *Conclusions*: The Breslow thickness >2 mm, high Clark level, high mitotic rate and ulceration are the most important prognostic factors for lymph nodal spread in cutaneous melanomas. However, some melanomas without these clinical-pathological features can have an unexpected, aggressive evolution, which entails the necessity of close and prolonged clinical follow-up of patients, including those with lesions considered without risk.

## 1. Introduction

In the last decades, the incidence of cutaneous melanoma increased significantly more than that of other neoplasms. This is the result of increased ultraviolet (UV) radiation exposure and/or a higher diagnosis rate due to a more careful observation of cutaneous lesions [1,2]. A family history of melanoma and a high number of nevi are equally important risk factors for the development of this type of cancer [3,4].

In 2020, according to the Global Cancer Observatory, 324.635 new cases of cutaneous melanoma were recorded worldwide, as well as 57.043 deaths caused by this disease [5]. A total of 105.172 new cases of cutaneous melanoma were diagnosed in North America. An increased incidence was observed in Australia and New Zealand. In Europe, the incidence varied between countries, the highest being in the West and the North, while the lowest was recorded in the South European countries, probably because people from the Northern countries have a lighter skin tone, but also because of the different manners of exposure to UV radiations (intermittent vs. chronic) [1,5]. In Romania, 1547 new cases of cutaneous melanoma were discovered in the same year, with a mortality of 502 cases [6].

The prognosis of melanoma depends on a series of clinical and pathological parameters, some of them being well known and validated by numerous studies. For other parameters, there are no consistent data regarding their role as prognostic factors in the evolution of patients with melanoma.

In this context, the present study proposes a retrospective analysis of clinical and pathological characteristics of cutaneous melanomas, as well as their correlations with the identification of parameters that have a prognostic role in the evolution and lymph nodal spread of this malignant tumor. Knowing these clinical, evolutive, and prognostic parameters can help the implementation of prevention programs and early diagnosis of melanoma, which in turn would lead to the prolonged survival of these patients. 

## 2. Materials and Methods

We performed a retrospective observational study, in a single institution, on the cases of invasive cutaneous melanoma, diagnosed and staged on surgical resection specimens, excised in the Departments of Surgery of the “Pius Brînzeu” County Clinical Emergency Hospital from Timișoara, on a period of 6 years. Resected specimens were processed by standard paraffin embedding in the Pathological Anatomy Service of the same hospital and examined in the usual hematoxylin–eosin stain.

We performed a computerized search for pathology reports that included the keyword “melanoma” in the database of the Pathological Anatomy Service and in the time interval between 1 January 2016 and 31 December 2021. We established the following inclusion and exclusion study criteria:

Inclusion criteria:

- Patients with invasive cutaneous melanoma;
- Patients with invasive cutaneous melanoma who were evaluated for the status of sentinel lymph nodes (SLN)/loco-regional lymph nodes (LN);

Exclusion criteria:

- Patients with in situ cutaneous melanoma;
- Patients with melanoma in another location than the skin;
- Patients with invasive cutaneous melanoma that were not evaluated for the status of SLN/loco-regional LN;
- Patients diagnosed with cutaneous melanoma in another medical unit who presented to our institution for the evaluation of the SLN/loco-regional LN status;
- Patients with metastases from melanoma diagnosed in another medical institution.

From the histopathological data of patients, we selected the following parameters: sex (male/female); the age of patients (≤50 years; >50 years); localization of the lesions; histological type; Breslow tumor thickness (pT), Clark level; SLN/loco-regional LN status (present/absent metastasis/metastases); satellite/in-transit metastasis (SINTM) (absent/present); lymphovascular invasion (LVI) (absent/present); perineural invasion (PNI) (absent/present); ulceration of the epidermis overlying the tumor (absent/present); mitotic rate; tumor-infiltrating lymphocytes (TILs), tumor regression (absent/present).

The pTNM (Tumor, Nodal, and Metastasis) staging was performed at the time of diagnosis according to the American Joint Committee on Cancer (AJCC) Cancer Staging Manual, 8th ed., 2017. The cases diagnosed and staged before the implementation of this system were re-evaluated and staging was updated. 

The pT parameter was established depending on the thickness of the tumor (Breslow thickness measured from the surface of the granular layer of the epidermis to the point of maximum depth of the tumor) as follows: pT1: <1 mm, T2: 1.01–2.0 mm, T3: 2.01–4.0 mm, and T4: >4 mm. For some of the statistical analyses, we classified the cases according to the thickness of the tumor into 2 categories: tumors with ≤2 mm thickness (pT1–pT2) and tumors >2 mm (pT3–pT4).

Depending on the Clark level, the cases were allocated into two categories: melanomas with the invasion of the entire papillary dermis (Clark level II–III) and melanomas with deep invasion down to the reticular dermis or the hypodermic tissue (Clark level IV–V). 

Mitotic rate (the number of mitoses per mm^2^) was assessed using the hot spot method, which consisted of identifying the area of the dermis with the highest number of mitoses, followed by counting the mitoses from the adjacent fields until an area of 1 mm^2^ was obtained (approximately 5 high power fields (HPF)). The cases were classified into 3 categories: 0–1 mitoses/mm^2^, 2–4 mitoses/mm^2^, and ≥5 mitoses/mm^2^.

A tumor-infiltrating lymphocytes (TILs) parameter was evaluated regarding both the extent and intensity as absent, non-brisk, and brisk. In brisk TILs, lymphocytes were present along the entire invasive growth base or diffusely infiltrated the entire tumor. In non-brisk TILs, one or more foci of intratumor lymphocytes were present. 

Tumor regression was assessed as being present when the following were identified: variable decrease in the number of melanoma cells in the dermal component of the tumor, mononuclear inflammatory infiltrate and melanophages in dermis, dermal fibrosis, accompanied by increased vascularity of the dermis. 

Satellite metastases were defined by the presence of nests or nodules (macroscopic or microscopic) in the first 2 cm from the primary melanoma, while in-transit metastases were characterized by the presence of cutaneous or subcutaneous metastases at over 2 cm from the primary melanoma, but not further than the regional lymph nodes.

### Statistical Analysis

The statistical analysis of the evaluated parameters was performed using the functions of Microsoft Office Excel 2007 (Microsoft Corp., Redmond, WA, USA) and GraphPad Prism software, Version 9.5.1 (GraphPad Software Inc., San Diego, CA, USA). To analyze the correlations and/or differences between various parameters, we used Chi-square and Fisher’s exact tests. The results were considered statistically significant when the value of *p* was <0.05.

## 3. Results

We identified 79 patients who met the inclusion criteria of our study. Demographic, clinical, and pathological data of patients included in the study group are presented in Table 1.

This study included 29 men (36.7%) and 50 women (63.3%) with a mean age at the time of diagnosis of 59.36 years (between 30 and 84 years old). The mean age of men was 60.8 years, while for the women it was 58.5 years. A total of 56 patients (70.9%) were over 50 years old. A number of 16 patients ≤50 years old (69.56%) were women (the ratio of women/men was 2.28/1). Over 50 years of age, the ratio of women/men was 1.54/1.

The most frequent anatomical site of melanomas was on the lower limbs: 28 cases (35.4%), followed by the trunk: 21 cases (26.6%), upper limbs: 16 cases (20.3%), head and neck: 13 (16.4%) cases. In 1 patient (1.3%), the localization of the tumor was not specified. We did not observe statistically significant differences between sexes regarding the localization of the lesions, but we did note a larger number of melanomas on the lower limbs of women (21/79 cases versus 7/79 cases) and on the trunk (13/79 versus 8/79 cases).

According to histological type, the cases were divided as follows: 41 (51.9%) superficial spreading melanomas (SSM), 24 (30.4%) nodular melanomas (NM), 11 (13.9%) acral melanomas (AM), and 3 (3.7%) “other types” (this category included 2 melanomas arising from a blue nevus and one nevoid melanoma).

**Figure 1:**
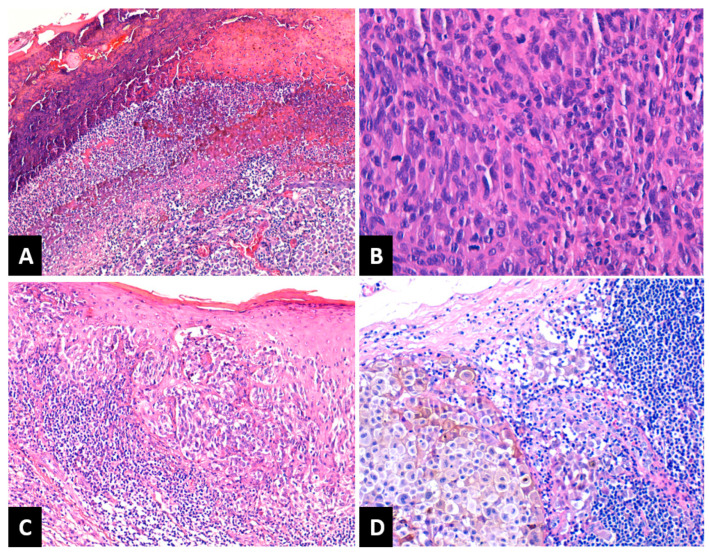
(**A**) NM with ulceration of the epidermis overlying the tumor: fibrin deposition and neutrophils. HE × 100; (**B**) NM with high mitotic rate. HE × 400; (**C**) SSM with brisk TILs. HE × 100; (**D**) Lymph nodal metastasis of melanoma with melanic pigment deposition. HE × 200; NM: nodular melanoma; SSM: superficial spreading melanoma; TILs: tumor-infiltrating lymphocytes.

Depending on Breslow tumor thickness (pT), the melanomas were classified as follows: 14 cases (17.7%) as pT1, 19 cases (24%) pT2, 15 cases (19%) pT3 and 31 cases (39.3%) pT4. A number of 24 melanomas (30.4%) had a Clark level of II–III and 55 melanomas (69.6%) had an IV–V Clark level. LVI was present in eight patients (10.1%). PNI was recorded in six patients (7.5%). Tumor ulceration was present in 47 melanomas (59.5%), Figure 1A. Regarding the mitotic rate, the cases were classified as follows: 14 cases (17.7%) in the category with 0–1 mitoses/mm^2^, 27 cases (34.2%) had 2–4 mitoses/mm^2^, and 38 cases (48.1%) were in the category ≥5 mitoses/mm^2^ (Figure 1B). After assessing TILs, the cases were grouped as follows: absent TILs—6 cases (7.6%), non-brisk TILs—47 cases (59.5%), and brisk TILs—26 cases (32.9%), Figure 1C. SINTM were observed in 18/79 (22.8%) patients and more frequently in men (15/18 cases versus 3/18 cases). Tumor regression was present in 35 melanomas (44.3%) and absent in 44 cases (55.7%). When present, regression involved less than 75% of the lesion in all cases. A number of 34 patients (43.1%) presented lymph node metastases (Figure 1D).

We did not observe statistically significant differences between men and women or between patients ≤50 and >50 years old regarding the histological type of melanoma, pT parameter, loco-regional lymph nodes status, the presence of satellite/in-transit metastases, LVI or PNI, tumor ulceration, mitotic rate, and TILs.

The analysis of the correlations between the pT parameter and the histological type of melanoma highlighted that the majority of NM were pT3–pT4 (22/79 cases) and only 2/79 cases were pT1–pT2, while SSM ≤ 2 mm thick (pT1–pT2) were more numerous (27/79 cases pT1–pT2 versus 14/79 cases pT3–pT4) (*p* < 0.0001). A number of 28/79 pT3–pT4 melanomas (35.4%) and 6/79 pT1–pT2 melanomas (7.6%) presented lymph node metastases (*p* = 0.0002). Most pT3–pT4 melanomas (38/79 cases) were ulcerated (*p* < 0.0001). A number of 34/79 pT3–pT4 melanomas presented a mitotic rate of ≥5 mitoses/mm^2^ (*p* < 0.0001). The pT parameter correlated positively with Clark level (*p* < 0.0001), LVI (*p* = 0.0181), PNI (*p* = 0.0377), SINTM (*p* = 0.0157), and TILs (*p* = 0.0106), Table 2.

After assessing the correlations between the status of loco-regional lymph nodes and other clinical-pathological parameters, we observed that the majority of melanomas with lymph node metastases (Figure 1) were ulcerated (26/34 cases) (*p* = 0.0107) and had a mitotic rate of ≥5 mitoses/mm^2^ (22/34 cases) (*p* = 0.0044). Most melanomas with LVI (7/8 cases) presented lymph node metastases (*p* = 0.0568). As mentioned above, loco-regional LN status correlated positively with the pT parameter (*p* = 0.0002) and with Clark level (*p* = 0.0026). Most patients under 50 years old did not have lymph node metastases (16/23 cases), as seen in Table 3.

We observed a statistically significant correlation between ulceration and the histological type of melanoma (*p* = 0.023). Most NM were ulcerated (20/24 cases versus 4/24 cases). SSM without ulceration were slightly more numerous (22/41 cases versus 19/41 cases). The majority of ulcerated melanomas had a III–IV Clark level (39/47 cases) (*p* = 0.0026), high Breslow tumor thickness (38/47 cases pT3–pT4) (*p* < 0.0001), and a mitotic rate of ≥5 mitoses/mm^2^ (32/47 cases) (*p* < 0.0001), as seen in Table 4.

A mitotic rate of ≥5 mitoses/mm^2^ was observed in NM (23/38 cases, *p* < 0.0001), and in those with a III–IV Clark level (35/38 cases) (*p* < 0.0001). As we showed above, mitotic rate correlated positively with the pT parameter (*p* < 0.0001), with loco-regional LN status (*p* = 0.0044) and tumor ulceration (*p* < 0.0001), as seen in Table 4.

Analyzing the features of melanomas with SINTM, we observed that they all presented a III–IV Clark level (18/18 cases) (*p* = 0.0008); most of them had high Breslow thickness (16/18 cases pT3–pT4) (*p* = 0.0013), were ulcerated (15/18 cases) (*p* = 0.0277), and associated LN metastases (12/18 cases) (*p* = 0.0302). We also noted a positive correlation between the presence of SINTM and LVI (5/18 cases) (*p* = 0.0132) and PNI (4/18 cases) (*p* = 0.0221), as seen in Table 2.

Regarding the tumor regression, it was absent especially in pT3-T4 melanomas (30/44 cases, *p* = 0.0659), ulcerated melanomas (27/44 cases, *p* = 0.8183), melanomas with a mitotic rate ≥5 mitoses/mm^2^ (25/44 cases, *p* = 0.1356), melanomas without SINTM (33/44 cases, *p* = 0.7879), and in melanomas without lymph nodes metastases (25/44 cases, *p* = 1). We did not observe a statistical correlation between the tumor regression and the other evaluated parameters, as seen in Table 2, Table 3 and Table 4.

## 4. Discussion

Melanoma is considered the most aggressive malignant tumor of the skin because of its unpredictable, often rapid evolution, with early distant metastases and poor prognosis. Cutaneous melanoma represents approximately 5% of all malignant tumors of the skin, but it contributes substantially to the mortality determined by cutaneous tumors [7,8].

Worldwide, in 2020, the incidence of cutaneous melanomas was somewhat higher in men (3.8 versus 3/100,000). In Oceania, the difference between sexes was higher (41.6 in men versus 30.5/100,000 in women). In Northern European countries, a higher incidence in women was observed (18.4 versus 17.4/100,000) [5]. Melanoma is one of the most common malignancies in patients under 50 years old [8], and although it is usually diagnosed at older ages, a higher frequency is observed in the 7th and 8th decades of life. In our study, most melanomas were diagnosed in female patients, with a women/men ratio of 1.72:1. The majority of patients were diagnosed in the 6–7th decades of life, but 29.1% of cases were observed at ages under 50 years old. There is a difference between patients’ gender regarding the age at diagnosis; in younger ages, melanomas are more frequent in women, while at older ages they are more frequent in men [1]. In our study, in patients under 50 years of age, melanomas were more frequent in women, with a ratio of women/men of 2.28/1. In patients over 50 years old, contrary to data from the literature [1,8], the lower frequency of melanomas in women was maintained, but with a women/men lower ratio (1.54/1).

Male gender, older age, and axial localization of lesions are associated with lymph node metastases, poor prognosis, and reduced survival [9,10,11,12,13,14]. Our results do not show statistically significant differences between men and women or between patients under 50 years or over 50 years regarding these parameters. Also, we did not notice a statistically significant association between the localization of tumors and the other parameters.

The status of loco-regional LN represents an important prognostic factor in cutaneous melanoma and its assessment is part of the standard procedure of staging. Moreover, patients with clinically negative LN, but with micro-metastases, will benefit from systemic therapy [15].

All these facts entail the assessment of parameters with a role in lymph node dissemination of cutaneous melanomas, in view of an adequate selection of patients whose SLN should be biopsied. Due to its prognostic importance, biopsy of SLN is recommended for all patients with melanomas thicker than 1mm, or with thickness ≥0.8 mm, and associated risk factors (presence of ulceration, mitotically active lesions, present LVI). Conversely, SNL biopsy is not recommended in patients with melanoma <0.8 mm and without ulceration [15,16,17,18].

In patients with thin melanomas, under 1 mm, the general positivity rate of SLN is approximately 5% [19,20], which led to significant controversies regarding the indication of SLN biopsy. Studies show that the most important predictive factors of SLN status are Breslow tumor thickness, ulceration, and mitotic rate, these parameters being mandatory to be reported in the histopathological sheets of patients [21,22,23,24]. Other parameters also associated with SLN metastases are age, sex, Clark level, presence of vertical growth phase, LVI, and SINTM [9,12,25,26].

Breslow tumor thickness represents the most important prognostic factor for the dissemination of cutaneous melanomas. Increased tumor thickness is associated with poor prognosis, higher risk of metastasis, and reduced survival [27,28].

Our study observed a statistically significant correlation between Breslow tumor thickness and loco-regional LN status (*p* = 0.0002). A percentage of 60.87% of pT3–pT4 melanomas presented lymph node metastases. Melanomas thicker than 2 mm were associated with a III–IV Clark level, the presence of ulceration, LVI and PNI; in turn, these characteristics are associated with high metastasis risk. We noted that 1/14 (7.14%) cases of thin melanomas, under 1mm, presented lymph node metastases. We mention that, in this case, other dissemination risk factors were not identified, such as tumor ulceration, mitotic rate, or lymphovascular invasion.

Kalady et al. show that approximately 15% of patients with thin melanomas will develop recurrences or metastases [29]. Clinical or pathological characteristics observed in lesions with dissemination risk are not present in all patients with aggressive tumors. To date, there are no clear selection criteria for patients, especially those with thin melanoma, with a high enough risk to justify SLN biopsy. The identification of risk factors for the aggressive behavior of some melanomas is still a challenge and early detection of new markers that will help in selecting the patients, which should receive SLN biopsy, is necessary [29,30].

Tumor ulceration is an important prognostic factor, being a part of the AJCC staging system, and represents the third most powerful predictive factor for survival, after tumor thickness and mitotic rate [31].

It is important to differentiate real ulceration from the separation between the epidermis and underlying layers, as a result of sectioning or processing artifacts. The presence of tissue reaction to the loss of the epidermis, with fibrin deposition and acute inflammation are aspects that show the presence of real ulceration. Ulceration is defined by loss of the entire epidermis, including the absence of stratum corneum and basal membrane, fibrin, and neutrophil deposition, as well as thinning, effacement, or reactive hyperplasia of the adjacent epidermis, in the absence of trauma or recent surgical procedure [32].

The association between melanoma ulceration and an adverse prognosis was proven in many studies [13,27,33,34,35]. The presence of ulceration denotes a highly proliferative lesion, often associated with a high mitotic rate, but not all thick melanomas with important mitotic activity are ulcerated, which suggests the influence of other additional factors in the development of ulceration [36,37,38]. Because of the aggressive biological behavior associated with ulceration, a hypothesis was issued stating that this develops as a consequence of some intrinsic biological factors of the tumor, supporting dissemination and loss of epidermis integrity [36,39].

In their review, Barricklow et al. identified some of the factors involved in melanoma ulceration: male gender, age over 50 years old, presence of risk factors for systemic inflammatory conditions (diabetes, vitamin D deficiency, smoking), histological aspect, spindle cell lesions, vascular density, presence of angiotropism, some molecular alterations such as proteins involved in the epithelial–mesenchymal transition, proteins associated with the presence of antigens (increased Major Histocompatibility Complex (MHC) I and MX Dynamin-like GTPase 1 (MX1)), autophagy, alteration of microRNA, high macrophage and dendritic cell density, and increased expression of proinflammatory cytokines like Interleukin 6 [40]. 

In our study, we did not observe a statistically significant correlation between ulceration and the age of patients at diagnosis. However, we noted a statistically significant correlation between ulceration and tumor histology, NM being the subtype most frequently associated with ulceration, the latter being present in 83.33% of cases. In SSM, the number of ulcerated lesions was comparable to that of non-ulcerated lesions. The majority of pT3–pT4 melanomas were ulcerated, thus a strong association between ulceration and Breslow tumor thickness was observed. Also, ulceration was present, especially in melanomas with deep invasion and a Clark level III–IV. Ulceration was associated with lymph node metastases, 76.47% of patients with lymph node metastases presented ulcerated melanomas (*p* = 0.0107). These results, according to data from the literature, show that ulceration is the attribute of melanomas with aggressive behavior, both through their direct relationship with the dissemination risk, as well as indirectly through the association with thick lesions and deep invasion, which prove to have the highest risk of metastasis. 

In the 8th edition (2017) of AJCC staging for melanomas, mitotic rate is no longer considered a part of the staging criteria. Instead, tumor thickness greater than 0.8 mm and the presence of ulceration are recognized as more significant predictive factors for survival. Also, the most recent guides of the American Society of Clinical Oncology (ASCO)—Society of Surgical Oncology (SSO) for SLN biopsy eliminated mitotic rate as a risk factor for considering SLN biopsy [18]. Nevertheless, it is unanimously recognized that mitotic rate represents a very important prognostic factor in the evolution of melanomas and can still be taken into account when evaluating a patient’s general risk based on the assessment of clinical-pathological features and pathologists must report it in the histopathological evaluation sheets [9,41,42].

In a study carried out on 3661 melanoma patients, Azzola et al. showed that mitotic rate is the most important independent predictor factor for survival of patients with cutaneous melanoma, stronger than ulceration [43]. Thompson et al. showed that a high mitotic rate is associated with low survival probability, and that it is also the strongest prognostic factor, after tumor thickness [44]. In a retrospective study on a group of 149.273 melanoma patients, with an invasion depth less than 0.1 mm, Wheless et al. observed that increased mitotic rate is strongly associated with the positive status of loco-regional LN [45]. In the study conducted by Tejera-Vaqueriz et al., on 4249 patients with incipient melanoma, a mitotic rate of >2 mitoses/mm^2^ was associated with lymph node metastases and they recommend SLN biopsy in patients with thin melanomas, under 1mm thickness, and high mitotic rate [19]. 

The importance of mitotic rate in the prognosis of cutaneous melanoma was also highlighted by Kashani-Sabet et al. in a study on 5050 patients from two different geographic areas. These authors suggested that this parameter should be re-included in the staging process [46].

It is recommended that the mitotic rate be reported as the number of mitoses/1mm^2^. The number of high-power fields that are equivalent to 1mm^2^ needs to be adjusted individually for each microscope [15]. Although the cut-off of the mitotic rate varies in different studies, the risk of recurrence and lymph node metastases, as well as decreased survival, are linked to the increase in the mitotic rate, especially in melanomas with incipient invasion [43].

In the present study, we observed high mitotic activity in NM, in melanomas with increased Breslow thickness, and high Clark level. Moreover, we noted a positive, statistically significant correlation between mitotic rate and the status of loco-regional LN (*p* = 0.0044), more important than the correlation between ulceration—the status of loco-regional LN. However, a large percentage of melanomas (29.2%) with a rate of ≤4 mitoses/mm^2^ presented lymph node metastases, this aspect highlighting the importance of this parameter in assessing the risk of dissemination of melanomas. These results support the idea that mitotic rate is a parameter that can contribute to a more accurate definition of risk categories and suggest its re-introduction into the pT staging system of melanoma. 

Regarding the behavior of melanomas according to their histology, NM are associated with a poor prognosis, these tumors showing histopathologic aggressivity parameters (higher Breslow tumor thickness, higher Clark level, presence of ulceration, and lymphovascular invasion) [47]. Susok et al. observed that NM is not a significant independent predictor factor for recurrence or disease-related death [48]. When important prognostic factors such as tumor thickness and ulceration are taken into account, the histological type of melanoma does not have great prognostic significance [9]. 

In our study, NM had more aggressive local behavior. Most of them had a thickness greater than 2 mm, were ulcerated, and presented an increased mitotic rate. However, we did not observe a direct statistically significant correlation between histological type and the presence of lymph node metastases.

Although Clark level has lost its meaning and is no longer found among the parameters recommended by the international guidelines for reporting, in some of the most important and recent studies that investigated predictive factors for dissemination in SLN in patients with thin melanomas, Clark level was an independent predictor factor for the presence of metastases in SLN [26].

In this study, Clark level was associated with the status of SLN/loco-regional LN (*p* = 0.0026) and is the second most important risk factor for lymph node metastases, after Breslow tumor thickness. About half of melanomas with a Clark level IV–V presented lymph node metastases and 16.6% of melanomas with a Clark level II–III associated lymph node metastases. We consider that Clark invasion level continues to be an important prognostic factor and should be mentioned in histopathological reports.

In our analysis, LVI and PNI were rather rarely identified during the histopathological examination of slides usually stained with hematoxylin–eosin. Their presence was observed in melanomas over 2 mm thick and melanomas with SINTM. Moreover, LVI was positively associated with lymph node metastases. 

In the 8th edition of the AJCC staging manual for melanomas, non-nodal regional metastases (microsatellites, satellite metastases, and in-transit metastases) are included in the pN category [31]. SINTM represents an adverse prognostic factor in patients with primary cutaneous melanoma and is associated with reduced survival [49]. Also, the presence of satellites is associated with metastases in SLN and local recurrence [50,51].

In our study, SINTM associated a III–IV Clark level, over 2 mm Breslow tumor thickness, ulceration, LVI, and PNI. Moreover, 66.6% of melanomas with SINTM presented lymph node metastases. These features show that SINTM melanomas have a more aggressive behavior and higher dissemination risk. 

Many studies showed that TILs represent a prognostic factor for patients with melanoma, with a correlation between different intensities or degrees of TILs and patients’ prognoses being observed [52]. Also, TILs are a predictive factor of SNL status and survival, patients with brisk TILs having an excellent prognosis [53]. Nevertheless, the role of TILs as a strong prognostic factor in melanoma is controversial. Contradictory results regarding their prognostic value may be the consequence of the immunophenotypic and functional heterogeneity of the intratumor lymphocytic infiltrate in melanoma [54,55]. Moreover, there are no clear criteria for the quantification of TILs in melanoma.

Our results highlighted that absent or non-brisk TILs were present, especially in melanomas over 2 mm thick. In the majority of melanomas (79.4%) with lymph node metastases, we observed absent or non-brisk TILs. These results show that reduced/absent TILs represent a negative prognostic factor in cutaneous melanoma.

Tumor regression is a parameter recommended to be reported in invasive cutaneous melanoma, but there is no uniformity in its definition and evaluation, leading to high interobserver variability. Some authors report the regression as present or absent, and others specify the regression stage [56]. According to the College of American Pathologists (CAP) protocol, regression should be reported as not identified, present—involving <75% of the lesion, present—involving ≥75% of the lesion, or undetermined [57]. This method supports the limitation of interobserver variability [58]. On the other hand, a cut-off of 75% is recommended as a limit for differentiating focal regression from extensive ones, because it has been observed, in some studies, that melanomas with ≥75% regression are associated with metastases [56,59].

The prognostic value of tumor regression in melanoma is controversial. Some studies show that regression is associated with the presence of lymph node metastases [60] and other results show the opposite [61,62].

In our study, tumor regression was not a prognostic factor for lymph node metastases. However, tumor regression was more frequent in melanomas with aggressive behavior (ulcerated melanomas, with an increased Breslow tumor thickness, and high mitotic rate), but without a statistical correlation between these parameters. Elaborate studies are needed to clarify the role of tumor regression in melanoma and its prognostic implications.

We emphasize that, beyond the common histological types, rare variants, such as melanoma arising from a blue nevus or nevoid melanoma, present in our study, are difficult to diagnose. Sometimes melanoma has an unusual histology that mimics nonmelanocytic neoplasms. In the present study, melanomas with ballooned or rhabdoid cells were recorded, but melanomas can have peculiar morphological patterns. Melanomas with carcinoid-like and paraganglioma-like patterns have also been reported [63].

### Study Limits

Our study has a retrospective character with the possibility of omitting some information. Also, it was carried out on a reduced number of cases. This study does not reflect the real incidence and prevalence of cutaneous melanoma cases during the investigated time. Another limitation of our study is the absence of information about the oncological treatment received and the subsequent evolution of patients, which would make it possible to correlate the results obtained with survival.

## 5. Conclusions

Our results show that Breslow tumor thickness represents the most important prognostic factor for lymph node dissemination in cutaneous melanoma, followed by Clark level, mitotic rate, and ulceration. Moreover, melanomas with lymphovascular invasion, with present satellite/in-transit metastasis and non-brisk TILs, show high risk for lymph node metastases. However, some melanomas without these clinicopathological features can have an unexpected aggressive evolution, which entails investigation of new biological markers associated with this behavior.

Also, we reiterate the necessity of careful and prolonged clinical follow-up of patients diagnosed with cutaneous melanomas, including those considered without dissemination risk, because of the unpredictable evolution of this neoplasm.

## Figures and Tables

**Table 1:** Clinical-pathological characteristics of the patients with invasive cutaneous melanoma.

Parameters		*n* = 79	%
Sex	Male	29	36.7
	Female	50	63.3
Age of diagnosis	Median age 59.36 years		
	≤50 years old	23	29.1
	>50 years old	56	70.9
Anatomic location	Head and neck	13	16.4
	Upper extremities	16	20.3
	Lower extremities	28	35.4
	Trunk	21	26.6
	Missing	1	1.3
Histologic subtypes	Superficial spreading melanoma	41	51.9
	Nodular melanoma	24	30.4
	Acral melanoma	11	13.9
	Other types	3	3.8
Clark level	II–III	24	30.4
	IV–V	55	69.6
Breslow tumor thickness	pT1 (<1 mm)	14	17.7
	pT2 (1.01–2 mm)	19	24
	pT3 (2.01–4 mm)	15	19
	pT4 (>4 mm)	31	39.3
Lymph node metastasis	Absent	45	56.9
	Present	34	43.1
Lymphovascular invasion	Absent	71	89.9
	Present	8	10.1
Perineural invasion	Absent	73	92.4
	Present	6	7.6
Tumor ulceration	Absent	32	40.5
	Present	47	59.5
Mitotic rate	0–1 mitoses/mm^2^	14	17.7
	2–4 mitoses/mm^2^	27	34.2
	≥5 mitoses/mm^2^	38	48.1
Satellite/in-transit metastasis	Absent	61	77.2
	Present	18	22.8
Tumor-infiltrating lymphocytes	Absent	6	7.6
	Non-brisk	47	59.5
	Brisk	26	32.9
Tumor regression	Absent	44	55.7
	Present	35	44.3

**Table 2:** Statistical analysis of the correlations between Breslow tumor thickness, satellites/in-transit metastasis, and the other prognostic parameters in the invasive cutaneous melanoma, using the Chi-square test and Fisher’s exact test.

Parameters		Breslow Tumor Thickness			Satellite/In-Transit Metastasis		
		pT1–pT2	pT3–pT4	*p*-Value	Absent	Present	*p*-Value
Sex	Male	12	17	>0.9999	19	10	0.0934
	Female	21	29		42	8	
Age of diagnosis	≤50 years old	13	10	0.1314	20	3	0.2449
	>50 years old	20	36		41	15	
Anatomic location	Head and neck	3	10	0.416	8	5	0.3628
	Upper extremities	6	10		12	4	
	Lower extremities	13	15		21	7	
	Trunk	10	11		19	2	
	Missing	1	0		1	0	
Histologic subtypes	Superficial spreading melanoma	27	14	<0.0001	34	7	0.6523
	Nodular melanoma	2	22		17	7	
	Acral melanoma	4	7		8	3	
	Other types	0	3		2	1	
Clark level	II–III	20	4	<0.0001	24	0	0.0008
	IV–V	13	42		37	18	
Breslow tumor thickness	pT1 (<1 mm)				14	0	0.0013
	pT2 (1.01–2 mm)				16	3	
	pT3 (2.01–4 mm)				14	1	
	pT4 (>4 mm)				17	14	
Lymph node metastasis	Absent	27	18	0.0002	39	6	0.0302
	Present	6	28		22	12	
Lymphovascular invasion	Absent	33	38	0.0181	58	13	0.0132
	Present	0	8		3	5	
Perineural invasion	Absent	33	40	0.0377	59	14	0.0221
	Present	0	6		2	4	
Tumor ulceration	Absent	24	8	<0.0001	29	3	0.0277
	Present	9	38		32	15	
Satellite/in transit metastasis	Absent	30	31	0.0157			
	Present	3	15				
Mitotic rate	0–1 mitoses/mm^2^	13	1	<0.0001	13	1	0.2501
	2–4 mitoses/mm^2^	16	11		21	6	
	≥5 mitoses/mm^2^	4	34		27	11	
Tumor-infiltrating lymphocytes	Absent	0	6	0.0106	3	3	0.1004
	Non-brisk	17	30		35	12	
	Brisk	16	10		23	3	
Tumor regression	Absent	14	30	0.0659	33	11	0.7879
	Present	19	16		28	7	

**Table 3:** Statistical analysis of the correlations between lymph nodal status and the prognostic parameters in invasive cutaneous melanoma, using the Chi-square test and Fisher’s exact test.

Parameters		Lymph Node Metastasis		
		Absent	Present	*p* Value
Sex	Male	15	14	0.4902
	Female	30	20	
Age of diagnosis	≤50 years old	16	7	0.2114
	>50 years old	29	27	
Anatomic location	Head and neck	8	5	0.8808
	Upper extremities	8	8	
	Lower extremities	16	12	
	Trunk	12	9	
	Missing	1	0	
Histologic subtypes	Superficial spreading melanoma	27	14	0.3555
	Nodular melanoma	11	13	
	Acral melanoma	6	5	
	Other types	1	2	
Clark level	II–III	20	4	0.0026
	IV–V	25	30	
Breslow tumor thickness	pT1 (<1 mm)	13	1	0.0002
	pT2 (1.01–2 mm)	14	5	
	pT3 (2.01–4 mm)	9	6	
	pT4 (>4 mm)	9	22	
Lymphovascular invasion	Absent	44	27	0.0182
	Present	1	7	
Perineural invasion	Absent	44	29	0.0792
	Present	1	5	
Tumor ulceration	Absent	24	8	0.0107
	Present	21	26	
Satellite/in-transit metastasis	Absent	39	22	0.0302
	Present	6	12	
Mitotic rate	0–1 mitoses/mm^2^	13	1	0.0044
	2–4 mitoses/mm^2^	16	11	
	≥5 mitoses/mm^2^	16	22	
Tumor-infiltrating lymphocytes	Absent	1	5	0.0302
	Non-brisk	25	22	
	Brisk	19	7	
Tumor regression	Absent	25	19	1
	Present	20	15	

**Table 4:** Statistical analysis of the correlations between ulceration, mitotic rate, and the other prognostic parameters in invasive cutaneous melanoma, using the Chi-square test and Fisher’s exact test.

Parameters		Ulceration			Mitotic Rate			
		Absent	Present	*p* Value	0–1 Mitoses/mm^2^	2–4 Mitoses/mm^2^	≥5 Mitoses/mm^2^	*p* Value
Sex	Male	13	16	0.6368	5	8	16	0.5872
	Female	19	31		9	19	22	
Age of diagnosis	<50 years old	12	11	0.2118	6	6	11	0.3861
	>50 years old	20	36		8	21	27	
Anatomic location	Head and neck	5	8	0.747	1	3	9	0.1256
	Upper extremities	6	10		1	7	8	
	Lower extremities	11	17		4	11	13	
	Trunk	10	11		7	6	8	
	Missing	1	0		1	0	0	
Histologic subtypes	Superficial spreading melanoma	22	19	0.023	12	18	11	<0.0001
	Nodular melanoma	4	20		1	0	23	
	Acral melanoma	4	7		1	7	3	
	Other types	2	1		0	2	1	
Clark level	II–III	16	8	0.0026	11	10	3	<0.0001
	IV–V	16	39		3	17	35	
Breslow tumor thickness	pT1 (<1 mm)	14	0	<0.0001	9	5	0	<0.0001
	pT2 (1.01–2 mm)	10	9		4	11	4	
	pT3 (2.01–4 mm)	4	11		1	4	10	
	pT4 (>4 mm)	4	27		0	7	24	
Lymph node metastasis	Absent	24	21	0.0107	13	16	16	0.0044
	Present	8	26		1	11	22	
Lymphovascular invasion	Absent	30	41	0.4625	14	24	33	0.3697
	Present	2	6		0	3	5	
Perineural invasion	Absent	29	44	0.6817	14	25	34	0.4455
	Present	3	3		0	2	4	
Tumor ulceration	Absent				12	14	6	<0.0001
	Present				2	13	32	
Mitotic rate	0–1 mitoses/mm^2^	12	2	<0.0001				
	2–4 mitoses/mm^2^	14	13					
	≥5 mitoses/mm^2^	6	32					
Satellite/in-transit metastasis	Absent	29	32	0.0277	13	21	27	0.2501
	Present	3	15		1	6	11	
Tumor-infiltrating lymphocytes	Absent	1	5	0.4649	0	2	4	0.4152
	Non-brisk	20	27		8	14	25	
	Brisk	11	15		6	11	9	
Tumor regression	Absent	17	27	0.8183	5	14	25	0.1356
	Present	15	20		9	13	13	

## Data Availability

All data generated or analyzed during this study are included in this published article and can be provided if needed or requested by the reviewer.
